# Around the Hospitals

**Published:** 1894-09-29

**Authors:** 


### Around the Hospitals.

[Notice to the Managers of Institutions.—Special space is now reserved for the insertion of notices of hospital meetings and festivals. The Editor requests that all notices may reach The Hospital Office, 428, Strand, at least one week before the date of each meeting.]

**Noble's Isle of Man General Hospital and Dispensary.**—This hospital has just completed another financial year, and the report issued is in many ways encouraging and satisfactory. In this case, as in that of so many other institutions in all parts of the country, the record of work is a steadily increasing one, and the last twelve months have witnessed a considerable augmentation of its sphere of usefulness. Owing to an increase in the nursing staff more accommodation is needed, the committee having been compelled, as it is, to rent rooms outside the hospital. Efforts are being made to secure ground or a building for this purpose, and it is satisfactory to find that the difficulty in doing this lies not in want of funds but in procuring house or land within the desired area. The present staff is sufficiently large to admit of private nursing being undertaken, 43 private cases having been attended during last year, the services of the nurses being greatly appreciated, and resulting in increased profit to the hospital. The total receipts for the year 1893-4 show a considerable increase, though some of the permanent subscriptions have fallen off. We trust this latter fact is but temporary, however, and that next year's report will present increased subscriptions as well as unsolicited donations. We are glad to see that an increase has to be noted in the subscriptions from employers and their workmen, proof positive that the hospital is popular amongst that portion of the population for whose benefit it exists. The experiment of a Hospital Saturday collection was tried this year, and proved eminently satisfactory as an addition to the funds, the sum gained amounting to £100. Very cordial votes of thanks are accorded to the honorary and medical staffs, and to the matron and nurses for their unremitting care and attention.

**The British Lying-in Hospital.** — This useful charity, the oldest of its kind in London, and situated in one of the poorest parts of the City, continues to be much appreciated, and accomplishes its work with success. The death-rate during five years has been only one in 190, in spite of the impoverished and unhealthy condition of a large number of the patients. Besides the benefit it confers on the patients, it acts as an important training school for midwives and nurses. Economy, having regard at the same time to sufficiency, has been carefully studied in the expenditure, and a large reduction in maintenance has been made. The sanitary arrangements of the hospital have been improved, and other improvements are contemplated. Increased subscriptions are appealed for. During the year 189 in-patients were treated and 420 patients attended in their own homes. The ordinary income amounted to £1,506, the ordinary expenditure to £1,426.

